# Assessing unrealised potential for organ donation after out-of-hospital cardiac arrest

**DOI:** 10.1186/s13049-021-00924-z

**Published:** 2021-07-28

**Authors:** Andrew Fu Wah Ho, Timothy Xin Zhong Tan, Ejaz Latiff, Nur Shahidah, Yih Yng Ng, Benjamin Sieu-Hon Leong, Shir Lynn Lim, Pin Pin Pek, Han Nee Gan, Desmond Renhao Mao, Michael Yih Chong Chia, Si Oon Cheah, Lai Peng Tham, Marcus Eng Hock Ong

**Affiliations:** 1 Department of Emergency Medicine, Singapore General Hospital, Singapore, Singapore; 2 Pre-hospital and Emergency Research Center, Duke-NUS Medical School, Singapore, Singapore; 3 Emergency Department, Tan Tock Seng Hospital, Singapore, Singapore; 4 Lee Kong Chian School of Medicine, Nanyang Technological University, Singapore, Singapore; 5 Emergency Medicine Department, National University Hospital, Singapore, Singapore; 6 Department of Cardiology, National University Heart Centre, Singapore, Singapore; 7 Health Services & Systems Research, Duke-NUS Medical School, Singapore, Singapore; 8 Accident & Emergency, Changi General Hospital, Singapore, Singapore; 9 Department of Acute and Emergency Care, Khoo Teck Puat Hospital, Singapore, Singapore; 10 Emergency Medicine Department, Ng Teng Fong General Hospital, Singapore, Singapore; 11 Children’s Emergency, KK Women’s and Children’s Hospital, Singapore, Singapore

**Keywords:** Uncontrolled donations after cardiac death, Organ donation, Extracorporeal cardio-pulmonary resuscitation, Pan-Asian resuscitation outcomes study, Cardiac arrest, Transplant

## Abstract

**Background:**

Organ donation after brain death is the standard practice in many countries. Rates are low globally. This study explores the potential national number of candidates for uncontrolled donations after cardiac death (uDCD) amongst out-of-hospital cardiac arrest (OHCA) patients and the influence of extracorporeal cardiopulmonary resuscitation (ECPR) on the candidacy of these potential organ donors using Singapore as a case study.

**Methods:**

Using Singapore data from the Pan-Asian Resuscitation Outcomes Study, we identified all non-traumatic OHCA cases from 2010 to 2016. Four established criteria for identifying uDCD candidates (Madrid, San Carlos Madrid, Maastricht and Paris) were retrospectively applied onto the population. Within these four groups, a condensed ECPR eligibility criteria was employed and thereafter, an estimated ECPR survival rate was applied, extrapolating for possible neurologically intact survivors had ECPR been administered.

**Results:**

12,546 OHCA cases (64.8% male, mean age 65.2 years old) qualified for analysis. The estimated number of OHCA patients who were eligible for uDCD ranged from 4.3 to 19.6%. The final projected percentage of potential uDCD donors readjusted for ECPR survivors was 4.2% (Paris criteria worst-case scenario, *n* = 532) to 19.4% of all OHCA cases (Maastricht criteria best-case scenario, *n* = 2428), for an estimated 14.3 to 65.4 uDCD donors per million population per year (pmp/year).

**Conclusions:**

In Singapore case study, we demonstrated the potential numbers of candidates for uDCD among resuscitated OHCA cases. This sizeable pool of potential donors demonstrates the potential for an uDCD program to expand the organ donor pool. A small proportion of these patients might however survive had they been administered ECPR. Further research into the factors influencing local organ and patient outcomes following uDCD and ECPR is indicated.

## Background

Since the first successful kidney transplant at the Peter Bent Brigham Hospital in 1952, organ transplantation has significantly enhanced the survival and quality of life of countless end-stage organ failure patients [1, 2]. Many countries including Singapore, a first world city-state with a population of 5.7 million [3] have relied on living donors and donation after brain death (DBD), which follows the 1968 Harvard Ad Hoc Committee’s definition of death as the irreversible cessation of brain function [4]. However, the demand for organs has persistently outstripped supply, fueled by aging populations, changing behavioral determinants of health, and surges in the prevalence of metabolic diseases associated with organ failure such as diabetes mellitus [1, 5–9]. This shortage is especially evident in developed countries such as Singapore, which has one of the highest incidences of treated end-stage renal disease in the world – 333 patients per million population (pmp) [10], but low organ donation rates (deceased organ donation rate of 6.6 DBD donors pmp in 2017) [2, 11, 12].

In response, several countries such as France, Spain and the Netherlands have turned to donation after circulatory death (DCD) to meet the demands for organ transplantation [13–16]. Although case studies from these countries have demonstrated poorer outcomes for liver transplants from DCD compared to DBD donors, there are comparable outcomes between DCD and DBD for kidney and lung transplants [1, 4, 17, 18]. DCD presently results in approximately 2.1 organs transplanted per donor, translating to 10.8 quality-adjusted life-years (QALYs) gained [19–21]. This presently constitutes half of all deceased organ donors in the United Kingdom [2] and is estimated to supply 61% of all donated organs in the United States [5, 17, 22]. DCDs can be categorized according to the modified Maastricht criteria into either controlled DCD (cDCD) where organ donation follows planned withdrawal of life support or uncontrolled DCD (uDCD) where donation follows unplanned circulatory death after unsuccessful cardiopulmonary resuscitation attempts [23].

Improvements in pre-hospital emergency care and bystander interventions have resulted in increasing proportions of out-of-hospital cardiac arrest (OHCA) cases with short no-flow or low-flow times (Pre-hospital/Emergency Department Return of Spontaneous Circulation (ED ROSC) rates at 18.3% in 2001–04 vs 23.8% in 2015–16) [24–26]. In the hospital setting, the progressive adoption of extracorporeal cardiopulmonary resuscitation (ECPR) characterized by the use of extracorporeal membrane oxygenation (ECMO) during CPR not only serves to optimize the outcomes of OHCA patients but may also become a bridging intervention towards uDCD for OHCAs who do not survive [27]. These advancements allow for prolonged organ viability despite cardiac death and position uDCD as a potential source of transplant organs [2, 22, 28]. This study aims to explore the potential national numbers for uDCD amongst OHCA cases using Singapore as a case study and ascertain the influence of ECPR on potential OHCA survivors. We hypothesize that there is an unrealized pool of uDCD donors amongst OHCA cases and that although several OHCA cases may survive neurologically intact with the administration of ECPR, these numbers are low.

## Methods

### Study setting and population

Singapore is a rapidly aging Asian city with a life expectancy of 83.1 years and a population of 5.7 million in 2019 [3]. The incidence of OHCA has been rising every year, with crude incidence of 26.5 pmp in 2011 to 44.6 per 1000 pmp in 2016. While an increasing proportion of these survived (19.9% survived to admission, 6.5% survived to discharge in 2016) [26], a large majority did not survive to admission. All of Singapore’s 18 hospitals (8 public, 10 private) provide deceased donors for solid organ transplantation. Kidney and liver transplantations are performed at two public academic medical centers (AMCs): Singhealth Duke-National University of Singapore (SDNUS) and the National University Health System (NUHS), while other forms of organ transplantations are centralized at either of the two AMCs (heart and lung transplantations at SDNUS, and pancreas transplantations at NUHS) [29]. Out-of-hospital cardiac arrest was defined as: absence of pulse, unresponsiveness and apnea, regardless of etiology and method of arrival [30, 31]. We excluded patients who attained ROSC in ED as they would have been admitted to intensive care units (ICUs) for post-resuscitative care.

### Study design

This was a retrospective, nationwide, multi-center cohort study of consecutive OHCA cases presenting to all public restructured hospitals in Singapore from 2010 to 2016. As uDCD is a relatively novel practice with few active programs worldwide, there are no accepted criteria for selecting candidates for uDCD, with many components being established based on empirical grounds [17]. However, several standardized protocols have been proposed [17, 32, 33]. All major articles on uDCD were reviewed, and four established protocols were selected and applied onto the study population – the Madrid [13], San Carlos Madrid [14], Maastricht [15], and Paris criteria [16]. Although each protocol differed in terms of individual criteria: age, comorbidities, and time cutoffs from collapse to CPR initiation (Table 1), all emphasized the spirit of exhausting all practicable resuscitation efforts as per international standards and evidence-based termination-of-resuscitation rules [17]. This yielded four different hypothetical populations of potential uDCD donors. Both the Maastricht and Paris criteria included an option to accept cases with signs of infection (best-case scenario) or exclude them from being uDCD donors (worst-case scenario). As the registry used did not collect specific data on signs of infection, we computed two extreme scenarios: assuming all OHCA of unknown causes were due to infection (worst-case scenario), and assuming none of them were due to infection (best case scenario). This would provide a range of estimates within which the population parameter would lie. The potential use of ECPR to preserve end-organ perfusion may serve to act as both a life-saving intervention, as well as an eventual bridge to uDCD in the event of unsuccessful resuscitation. As such, a condensed ECPR eligibility criteria (age between 18 and 75 years, no severe comorbidities, cardiac etiology, time to CPR initiation < 5 min, shockable rhythm, no ROSC within 20 min) as well as an estimated ECPR neurologically intact survival rate of 12.3% derived by Sakamoto et al. [34] was applied to these four populations. This allowed us to extrapolate for possible OHCA survivors (and therefore not suitable to be uDCD donors), had ECPR been administered in the field.

**Table 1:** Constituent Factors and Projected Donor Numbers per uDCD Criteria

		uDCD Criteria					
		Madrid^13^	San Carlos, Madrid^14^	Maastricht^15^		Paris^16^	
				Best-case	Worst-case	Best-case	Worst-case
**Factors**	Age (years)	18–60	7–55	< 65		18–55	
	Witnessed OHCA	Yes	Yes	Yes		Yes	
	Cardiac Arrest to CPR Initiation (min)	< 15	< 10	< 45		< 30	
	Comorbidities for Exclusion	1. Unknown cause of death 2. Risk factors for HIV^†^ 3. Trauma to chest or abdomen	1. Unknown cause of death 2. Risk factors for HIV 3. Trauma to chest or abdomen	–	1. Infection	1. Hypertension 2. Diabetes 3. Cancer 4. Renal disease 5. Trauma to abdomen	1. Hypertension 2. Diabetes 3. Cancer 4. Renal disease 5. Trauma to abdomen 6. Infection
**Projected Patient Numbers**	Eligible for uDCD (% of total)	1202 (9.6)	660 (5.3)	2460 (19.6)	1987 (15.8)	648 (5.2)	544 (4.3)
	Eligible for ECPR (% of uDCD)	208 (17.3)	152 (23.0)	266 (10.8)	266 (13.4)	102 (15.7)	102 (18.8)
	ECPR Survivors^‡^	25	18	32	32	12	12
	Potential uDCD Donors (% of total)^††^	1177 (9.4)	642 (5.1)	2428 (19.4)	1955 (15.6)	636 (5.1)	532 (4.2)
	**Potential uDCD Donors (ppm/year)**^**§**^	**31.7**	**17.3**	**65.4**	**52.7**	**17.1**	**14.3**

^†^ HIV=Human Immunodeficiency Virus

^‡^ Refers to the estimated number of OHCA patients eligible for uDCD and ECPR that survive neurologically intact if ECPR was administered. This is obtained by multiplying the projected number of ECPR cases by 12.3% and rounding down to the nearest whole number (12.3% estimate derived from Sakamoto et al.^34^)

^††^ Refers to the final estimate of national uDCD donors. This is obtained by deducting ECPR survivors from the number eligible for uDCD

^§^ Assuming a resident population of 5.3 million across all 7 years of data (actual 2010 population 5.1 million; 2016 population 5.6 million). Current DBD donor rate is approximately 6.6 ppm/year

### Data source and collection

The Pan-Asian Resuscitation Outcomes Study Clinical Research Network (PAROS CRN) is an international prospective registry of OHCAs in the Asia-Pacific region [30, 35]. Established in 2010, it objectively reported consecutive OHCA events through standardized data definitions and collection methods across multiple cities to improve understanding of OHCA epidemiology in Asia [35]. Registry protocols have previously been described in literature [31]. The Centralised Institutional Review Board (2013/604/C) and Domain Specific Review Board (2013/00929) granted approval for this study with a waiver of patient informed consent. In this study, analysis was carried out on de-identified PAROS data collected prospectively in Singapore from Apr 1, 2010 to Dec 31, 2016.

Definitions for OHCA characteristics follow Utstein recommendations and include time sensitive OHCA data elements [36]. Patient demographics (age, gender, ethnicity), injury characteristics (cause of arrest, witnessed arrest, first arrest rhythm), pre-hospital management (bystander CPR, bystander automated external defibrillator [AED] use, pre-hospital defibrillation), and patient outcomes (pre-hospital/ED ROSC) were also prospectively recorded.

### Availability of data and materials

Data and analyses are not publicly available as they were used under license for the current study. Data is however available from the authors upon reasonable request.

### Statistical analysis

Data was reported as the mean (standard deviation [SD]) or median (interquartile range [IQR]) for continuous variables, and frequency (%) for categorical variables. Two-sample z-test was used to compare categorical variables by uDCD protocols. All statistical analyses were carried out via SPSS version 23 (IBM Corp).

## Results

Out of 12,546 patients with OHCA, 8530 (68.0%) patients did not survive to ED ROSC. Mean age was 65.2 years old (SD 18.5) and 64.8% were male. Sixty percent of cases were witnessed arrests (*n* = 7502), and asystole was the most common first arrest rhythm (48.7%, *n* = 6107), followed by Pulseless Electrical Activity (PEA), Ventricular Fibrillation (VF), and Ventricular Tachycardia (VT). 43.7% had bystander CPR (*n* = 5477), with a median time to CPR initiation of 15 min (IQR = 12.08), and 25.5% of cases had pre-hospital defibrillation (*n* = 3202) (Table 2). Compared to the population of OHCA cases, suitable uDCD donors were more likely to be male, non-Chinese OHCA patients, with VF as the first arrest rhythm as opposed to asystole (*p* < .05). Bystander AED and CPR, and pre-hospital defibrillation were correlated with suitability for uDCD (Table 2).

**Table 2:** Patient Demographics and OHCA Characteristics

	Total	Madrid Criteria	San Carlos, Madrid Criteria	Maastricht Criteria		Paris Criteria		***p***-value^††^
				Best-case	Worst-case	Best-case	Worst-case	
	*n* = 12,546	*n* = 1202	*n* = 660	*n* = 2460	*n* = 1987	*n* = 648	*n* = 544	
**Patient Demographics**								
Mean age in years (SD)	65.2 (18.5)	48.5 (9.0)	43.5 (9.8)	49.6 (12.8)	50.1 (11.8)	42.4 (9.2)	43.3 (8.6)	–
Male (%)	8125 (64.8)	975 (81.1)	526 (79.7)	1940 (78.9)	1653 (83.2)	563 (86.9)	494 (90.8)	**<.05**
Ethnicity (%)								
Chinese	8492 (67.7)	627 (52.2)	333 (50.5)	1327 (53.9)	1050 (52.9)	323 (49.8)	279 (51.3)	**<.05**
Malay	1961 (15.6)	276 (23.0)	150 (22.7)	540 (22.0)	430 (21.6)	112 (17.3)	86 (15.8)	**<.05** (excl. Paris)
Indian	1358 (10.8)	181 (15.0)	99 (15.0)	370 (15.0)	320 (16.1)	116 (17.9)	98 (18.0)	**<.05**
Others	735 (5.9)	118 (9.8)	78 (11.8)	223 (9.1)	187 (9.4)	97 (15.0)	81 (14.9)	–
Presence of comorbidities precluding organ donation^†^ (%)	8335 (66.4)	–	–	–	–	–	–	–
**OHCA Characteristics (%)**								
Witnessed Arrest	7502 (59.8)	–	–	–	–	–	–	–
First Arrest Rhythm^†^								
Asystole	6107 (48.7)	429 (35.7)	238 (36.0)	995 (40.4)	736 (37.0)	265 (40.9)	211 (38.8)	**<.05**
PEA	3519 (28.0)	307 (25.5)	156 (23.6)	700 (28.5)	542 (27.3)	140 (21.6)	104 (19.1)	>.05
VF	1908 (15.2)	371 (30.9)	201 (30.5)	589 (23.9)	563 (28.3)	194 (29.9)	186 (34.2)	**<.05**
VT	58 (0.5)	6 (0.5)	2 (0.3)	11 (0.4)	10 (0.5)	2 (0.3)	2 (0.4)	>.05
Bystander CPR	5477 (43.7)	690 (57.4)	442 (67.0)	1112 (45.2)	899 (45.2)	334 (51.5)	292 (53.7)	**<.05** (excl. Maastricht)
Median time to CPR initiation, min (IQR)	15.00 (12.08)	12.00 (8.08)	11.00 (10.00)	14.00 (10.23)	14.00 (10.00)	14.47 (9.45)	14.40 (9.19)	**–**
Bystander AED	399 (3.2)	74 (6.2)	52 (7.9)	112 (4.6)	97 (4.9)	41 (6.3)	39 (7.2)	**<.05**
Pre-hospital Defibrillation	3202 (25.5)	577 (48.0)	321 (48.6)	947 (38.5)	892 (44.9)	311 (48.0)	299 (55.0)	**<.05**

^†^ Examples include trauma, Acquired Immunodeficiency Syndrome (AIDS), signs of infection, and unknown causes of death

^‡^ PEA = pulseless electrical activity; VF = ventricular fibrillation; VT = ventricular tachycardia

^††^ Two-sample z-test was used for all statistical analyses

**Fig. 1:**
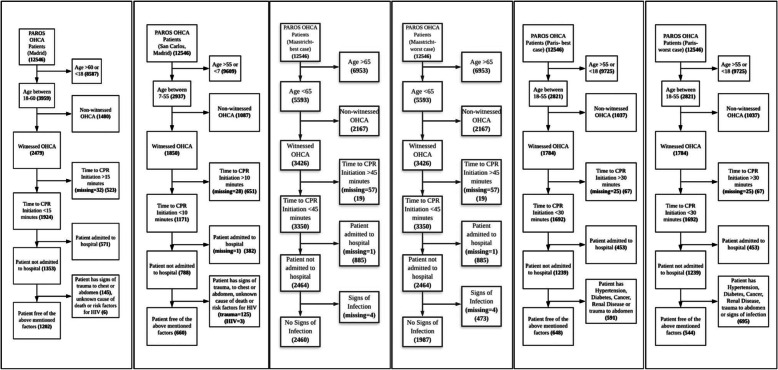
Flowcharts of Potential Donors, Singapore, 2010–2016, *n* = 12,546 OHCA cases. From left to right: Madrid [13], San Carlos Madrid [14], Maastricht best-case, Maastricht worst-case [15], Paris best-case, Paris worst-case [16]

Application of all four uDCD selection criteria resulted in six separate populations of OHCA patients (a best-case and worst-case scenario were obtained for both Maastricht and Paris criteria). The estimated number of OHCA patients who were eligible for uDCD ranged from 4.3 to 19.6% (*n* = 544–2460) (Fig. 1). Factoring in the possibility of successful ECPR with neurologically intact survival, a condensed ECPR eligibility criteria was then applied to each of the six sub-populations of potential uDCD donors to obtain the estimated number of OHCA cases which would have had ECPR administered in the emergency setting (*n* = 102–266). An ECPR neurologically intact survival rate of 12.3% [34] was then used to gauge the number of cases with successful ECPR (i.e. no longer suitable for uDCD as the donors have survived neurologically intact). The final projected percentage of potential uDCD donors readjusted for ECPR survivors is 4.2% (Paris criteria worst-case scenario, *n* = 532) to 19.4% of all OHCA cases (Maastricht criteria best-case scenario, *n* = 2428), translating to an estimated 14.3 to 65.4 uDCD donors per million population per year (pmp/year) (Table 1).

## Discussion

This secondary analysis of a nationwide prospective registry found that the number of OHCA patients who were candidates for uDCD ranged from 4.3 to 19.6% (*n* = 12,546). This is substantially higher than other studies which gave a range from 1.5 to 7.5% [5, 28], largely due to the more generous Maastricht criteria. We postulate that this is also due to rapidly improving pre-hospital emergency care and OHCA survival in Singapore. It is likely that these numbers will continue to increase as a result of increasing local OHCA incidence and advancements in OHCA resuscitation interventions. After adjusting for ECPR neurologically intact survivors, the revised range was 4.2 to 19.4%, translating to an estimated 14.3 to 65.4 uDCD donors pmp/year.

Male and Chinese OHCA patients made up the majority of OHCA cases (64.8, 67.7%) and also represented the majority of potential uDCD donors (79.7–90.8%, 49.8–53.9%). However, male and non-Chinese OHCA patients (specifically Malays and Indians) were significantly more likely to be potential uDCD donor candidates (*p* < .05). This is postulated to be because these subsets of OHCA patients were more likely to be younger and therefore more likely to meet the age criteria for uDCD [5, 13, 16]. The older average age of onset of OHCAs in Chinese patients also predisposes them to an increased prevalence of comorbidities that come with age, and likely resulted in their exclusion from uDCD candidacy [37]. As expected, bystander AED and CPR as well as pre-hospital defibrillation were correlated with suitability for uDCD (p < .05) as these pre-hospital interventions are associated with a reduced time to CPR initiation [24–26, 38] – one of our uDCD selection criteria.

Historically, Singapore has low organ donation rates with a deceased organ donation rate of 6.6 DBD donors pmp in 2017, low in comparison to other countries with opt-out policies such as Croatia (36.3 donors pmp) [2, 11, 12]. This is despite Singapore passing two separate legislations for organ transplantation to increase organ donation – the Medical (Therapy, Education and Research) Act (MTERA) in 1972 [7] and the Human Organ Transplant Act (HOTA) in 1988 [39]. MTERA is an *opt-in* policy which allows residents to donate organs or any other body parts for transplantation, education, or research upon death (of which only 1.3% have signed up) [29], while HOTA is a presumed consent policy for organ donation under which all mentally capable Singaporeans and permanent residents from age 21 years are organ donors (kidneys, heart, liver and corneas) unless they have *opted out* [2, 11, 29, 39, 40]. As a result, up to half of patients on the transplant waitlist eventually die or become too sick to undergo transplantation [40–42] and those who survive wait an average of 9 years before a suitable donor is found [42]. Suggested reasons for poor organ donation rates include low referral rates of potential donors by ICUs, early withdrawals of life support by family, reductions in traumatic brain injuries (TBIs) due in part to improvements in traffic and work safety measures and most importantly, cardiac arrest prior to the declaration of brain death [29, 41, 43].

Our results indicate that the implementation of uDCD protocols may substantially increase the number of potential organ donors from 6.6 DBD donors pmp currently, to 20.9–72 DBD/DCD donors pmp, countering the decreasing incidences of TBI and brain death. The potential to convert the inevitable death of one OHCA patient into QALYs for many others is tremendous, with an estimated 10.8 QALYs gained [21] from an average of 2.1 organs transplanted per DCD donor [19, 20]. The rising incidence of OHCAs also make uDCDs increasingly pertinent, and many sudden cardiac death patients are relatively young and healthy with no contraindications to organ donation [44]. Regardless of neuroprognostication, ECPR should be considered for OHCA patients as much as feasibly possible, as this may serve as an eventual bridge to uDCD in the event of unsuccessful resuscitation.

In countries such as Spain, DCDs now constitute 24% of all deceased donors [45], allowing them to reach an enviable target of 40 donors pmp [41]. However, attempts to improve organ donation rates must not compromise reasonable OHCA resuscitation efforts. Analysis of OHCA cases in Spain pre and post uDCD implementation have demonstrated no significant difference in ROSC rates, allaying concerns of any compromise in the care and treatment of potential organ donors [28, 46]. Further studies are indicated to optimize uDCD programs for local implementation to ensure the selection of only OHCA cases with zero prognosis for uDCD candidature. Public awareness and perception of such programs must also be considered and addressed prudently. This will minimize unfamiliarity or ill-informed opposition that may unnecessarily prolong the duration from cardiac death to transplantation, with consideration given to abstinence from coercion and pressure as well as provision of time and space to grieve. Other measures to improve organ donation rates such as revisions to transplant workflows to increase ICU referral rates of potential donors should also be considered.

### Limitations

Our study findings should be interpreted with the following limitations in mind. Firstly, as this is a multi-centre study spanning many years, data integrity and validity may be suboptimal. This was mitigated through pre-study standardisations of data definitions and data collection protocols, as well as the large sample size of the study. Secondly, as the registry did not collect specific data on signs of infection in OHCA patients, a proxy had to be utilised, where we assumed that all OHCAs of unknown cause were due to infection (worst-case scenario for the Maastricht and Paris criteria). Lastly, as this is a theoretical study, several models and calculations had to be extrapolated, limiting the extent that study results can be translated into actual practice. It also does not take into account several considerable real-life challenges and limitations. This includes the myriad of advanced resources required for ECPR and uDCD which ranges from equipment such as veno-arterial ECMO [46], to specialised manpower such as cardiothoracic surgeons, intensivists and perfusionists [41]. Ethical and legal considerations would also need to be deliberated and addressed appropriately [17, 41]. Additional analyses on the factors influencing the efficacy of uDCD and ECPR should be carried out.

## Conclusions

We demonstrated the potential numbers of candidates for uDCD among resuscitated OHCA cases using Singapore as a case study. This sizeable pool of potential donors demonstrates the potential for an uDCD program to expand the organ donor pool. A small proportion of these patients might survive neurologically intact had ECPR been administrated, and not be suitable for uDCD. Such attempts to improve organ donation rates must not compromise reasonable OHCA resuscitation efforts. Further research into the factors influencing organ and patient outcomes following uDCD and ECPR is indicated.

## Data Availability

Data and analyses are not publicly available as they were used under license for the current study. Data is however available from the authors upon reasonable request.

## Abbreviations

DCD: Donation after Circulatory Death
uDCD: Uncontrolled Donation after Circulatory Death
cDCD: Controlled Donation after Circulatory Death
OHCA: Out-of-hospital Cardiac Arrest
ECPR: Extracorporeal Cardiopulmonary Resuscitation
DBD: Donation after Brain Death
PMP: Patients per Million Population
QALY: Quality-adjusted Life-years
ED: Emergency Department
ROSC: Return of Spontaneous Circulation
ECMO: Extracorporeal Membrane Oxygenation
AMC: Academic Medical Center
SDNUS: Singhealth Duke-National University of Singapore
NUHS: National University Health System
ICU: Intensive Care Unit
PAROS: Pan-Asian Resuscitation Outcomes Study
CRN: Clinical Research Network
AED: Automated External Defibrillator
SD: Standard Deviation
IQR: Interquartile Range
PEA: Pulseless Electrical Activity
VF: Ventricular Fibrillation
VT: Ventricular Tachycardia
MTERA: Medical (Therapy, Education and Research) Act
HOTA: Human Organ Transplant Act
TBI: Traumatic Brain Injury

## References

[1] Kaufman BJ, Wall SP, Gilbert AJ, Dubler NN, Goldfrank LR (2009). Success of organ donation after out-of-hospital cardiac death and the barriers to its acceptance. Crit Care.

[2] Liu CWY, Ho VK, Liu JCJ. Is the human organ transplant act (HOTA) to blame? Addressing our organ shortage from a public policy perspective. Annals Acad Med Singapore 2017;46(10):392–4. 29177367

[3] Department of Statistics Singapore. Population Trends 2019. 2019. https://www.singstat.gov.sg/-/media/files/publications/population/population2019.pdf; 2019. Accessed 1 Sept 2020.

[4] Bendorf A, Kelly PJ, Kerridge IH, McCaughan GW, Myerson B, Stewart C, et al. An international comparison of the effect of policy shifts to organ donation following cardiocirculatory death (DCD) on donation rates after brain death (DBD) and transplantation rates. *PLoS ONE*. 2013;8(5); doi:10.1371/journal.pone.0062010

[5] Af Geijerstam P, Forsberg S, Claesson A, Djärv T, Jonsson M, Nordberg P (2019). Potential organ donors after out-of-hospital cardiac arrest during a ten-year period in Stockholm, Sweden. Resuscitation.

[6] Caplan AL (2016). Finding a solution to the organ shortage. CMAJ..

[7] Singapore Attorney-General’s Chambers. Medical (Therapy, Education and Research) Act (Chapter 175). 2014. https://sso.agc.gov.sg/Act/MTERA1972. Accessed 22 Aug 2020.

[8] United States Department of Health and Human Services. Organ Procurement and Transplantation Network. 2020. https://optn.transplant.hrsa.gov. Accessed 26 Aug 2020.

[9] Chin JJ. Mandated consent – not a viable solution for organ transplant in Singapore. Ann Acad Med Singap 2018;47(2);71–73. 29549373

[10] United States Renal Data System. Chapter 11: International Comparisons. 2018. https://www.usrds.org/media/1738/v2_c11_intcomp_18_usrds.pdf. Accessed 20 Aug 2020.

[11] Shum E, Chern A. Amendement of the human organ transplant act. Ann Acad Med Singap 2006;35:428–32. 16865196

[12] Channel News Asia Singapore. ‘Are you sure he is dead?’: Doctors struggle with families lack of understanding of HOTA. 2019. https://www.channelnewsasia.com/news/singapore/human-organ-transplant-act-doctors-families-understanding-11459284.

[13] Miranda-Utrera N, Medina-Polo J, Pamplona M, de la Rosa F, Rodríguez A, Duarte JM, Passas JB, Mateos-Rodríguez A, Díaz R, Andrés A (2013). Donation after cardiac death: results of the SUMMA 112 – hospital 12 de Octubre program. Clin Transpl.

[14] Nuñez JR, Del Rio F, Lopez E, Moreno MA, Soria A, Parra D (2005). Non-heart-beating donors: an excellent choice to increase the donor pool. Transpl Proc.

[15] Hoogland ERP, Snoeijs MGJ, van Heurn LWE (2010). DCD kidney transplantation: results and measures to improve outcome. Curr Opin Organ Transpl.

[16] Fieux F, Losser MR, Bourgeois E, Bonnet F, Marie O, Gaudez F, Abboud I, Donay JL, Roussin F, Mourey F, Adnet F, Jacob L (2009). Kidney retrieval after sudden out of hospital refractory cardiac arrest: a cohort of uncontrolled non heart beating donors. Crit Care.

[17] Domínguez-Gil B, Duranteau J, Mateos A, Núñez JR, Cheisson G, Corral E, de Jongh W, del Río F, Valero R, Coll E, Thuong M, Akhtar MZ, Matesanz R (2016). Uncontrolled donation after circulatory death: European practices and recommendations for the development and optimization of an effective programme. Transpl Int.

[18] Gagandeep S, Matsuoka L, Mateo R, Cho YW, Genyk Y, Sher L, Cicciarelli J, Aswad S, Jabbour N, Selby R (2006). Expanding the donor kidney pool: utility of renal allografts procured in a setting of uncontrolled cardiac death. Am J Transpl.

[19] Bellingham JM, Santhanakrishnan C, Neidlinger N, Wai P, Kim J, Niederhaus S, Leverson GE, Fernandez LA, Foley DP, Mezrich JD, Odorico JS, Love RB, de Oliveira N, Sollinger HW, D’Alessandro AM (2011). Donation after cardiac death: a 29-year experience. Surgery..

[20] Manara AR, Murphy PG, O’Callaghan G (2012). Donation after circulatory death. Br J Anaesth.

[21] Nunnick L, Cook DA (2016). Palliative ICU beds for potential organ donors: an effective use of resources based on quality-adjusted life-years gained. Crit Care Resusc.

[22] Lazzeri C, Bonizzoli M, Franci A, Cianchi G, Batacchi S, Ciapetti M, Fulceri GE, Rugna M, Peris A (2020). Out of hospital cardiac arrest and uncontrolled donation after circulatory death in a tertiary cardiac arrest center. Eur J Emerg Med.

[23] Kootstra G, Daemen JH, Oomen AP. Categories of non-heart-beating donors. Transpl Proc. 1995;27(5):2893–4. 7482956

[24] Lai H, Choong CV, Fook-Chong S, Ng YY, Finkelstein EA, Haaland B, Goh ES, Leong BS, Gan HN, Foo D, Tham LP, Charles R, Ong ME, PAROS study group (2015). Interventional strategies associated with improvements in survival for out-of-hospital cardiac arrests in Singapore over 10 years. Resuscitation..

[25] Ong MEH, Perkins GD, Cariou A (2018). Out-of-hospital cardiac arrest: prehospital management. Lancet..

[26] Blewer AL, Ho AFW, Shahidah N, White AE, Pek PP, Ng YY, Mao DR, Tiah L, Chia MYC, Leong BSH, Cheah SO, Tham LP, Kua JPH, Arulanandam S, Østbye T, Bosworth HB, Ong MEH (2020). Impact of bystander-focused public health interventions on cardiopulmonary resuscitation and survival: a cohort study. Lancet Pub Health.

[27] Domínguez-Gil B, Haase-Kromwijk B, Van Leiden H, Neuberger J, Coene L, Morel P (2011). Current situation of donation after circulatory death in European countries. Transpl Int.

[28] Navalpotro-Pascual JM, Echarri-Sucunza A, Mateos-Rodríguez A, Peinado-Vallejo F, Fernández del Valle P, Alonso-Moreno D (2018). Uncontrolled donation programs after out-of-hospital cardiac arrest An estimation of potential donors. Resuscitation.

[29] Kee T, Shridhar Ganpathi I, Sivathasan C, Kong S, Premaraj J, Anantharaman V (2018). Solid organ transplantation in Singapore. Transplantation..

[30] Ong MEH, Shin SD, Souza NNAD, Tanaka H, Nishiuchi T, Song KJ (2015). Outcomes for out-of-hospital cardiac arrests across 7 countries in Asia: the Pan Asian resuscitation outcomes study (PAROS). Resuscitation..

[31] Tan TXZ, Hao Y, Ho AFW, Shahidah N, Yap S, Ng YY (2019). Inter-hospital variations in resuscitation processes and outcomes of out-of-hospital cardiac arrests in Singapore. J Emerg Crit Care Med.

[32] Abboud I, Viglietti D, Antoine C, Gaudez F, Meria P, Tariel E, Mongiat-Artus P, Desgranchamps F, Roussin F, Fieux F, Jacob L, Randoux C, Michel C, Flamant M, Lefaucheur C, Pillebout E, Serrato T, Peraldi MN, Glotz D (2012). Preliminary results of transplantation with kidneys donated after cardiocirculatory determination of death: a French single-Centre experience. Nephrol Dial Transpl.

[33] Hoogland ERP, Snoeijs MGJ, Winkens B, Christaans MHL, van Heurn LWE (2011). Kidney transplantation from donors after cardiac death: uncontrolled versus controlled donation. Am J Transpl..

[34] Sakamoto T, Morimura N, Nagao K, Asai Y, Yokota H, Nara S, Hase M, Tahara Y, Atsumi T, SAVE-J Study Group (2014). Extracorporeal cardiopulmonary resuscitation versus conventional cardiopulmonary resuscitation in adults with out-of-hospital cardiac arrest: a prospective observational study. Resuscitation..

[35] Ong MEH, Shin SD, Tanaka H, Ma MHM, Khruekarnchana P, Hisamuddin N, Atilla R, Middleton P, Kajino K, Leong BSH, Khan MN (2011). Pan-Asian resuscitation outcomes study (PAROS): rationale, methodology, and implementation. Acad Emerg Med.

[36] Perkins GD, Jacobs IG, Nadkarni VM, Berg RA, Bhanji F, Biarent D, Bossaert LL, Brett SJ, Chamberlain D, de Caen AR, Deakin CD, Finn JC, Gräsner JT, Hazinski MF, Iwami T, Koster RW, Lim SH, Huei-Ming Ma M, McNally BF, Morley PT, Morrison LJ, Monsieurs KG, Montgomery W, Nichol G, Okada K, Eng Hock Ong M, Travers AH, Nolan JP, Aikin RP, Böttiger BW, Callaway CW, Castren MK, Eisenberg MS, Kleinman ME, Kloeck DA, Kloeck WG, Mancini ME, Neumar RW, Ornato JP, Paiva EF, Peberdy MA, Soar J, Rea T, Sierra AF, Stanton D, Zideman DA (2015). Cardiac arrest and cardiopulmonary resuscitation outcome reports: update of the Utstein resuscitation registry templates for out-of-hospital cardiac arrest. Circulation..

[37] Health Promotion Board, Singapore. Singapore Myocardial Infarction Registry Annual Report 2017, https://www.nrdo.gov.sg/docs/librariesprovider3/default-document-library/smir-web-report-2016_final.pdf?sfvrsn=0; 2018 (accessed 1 Sept 2020).

[38] Ng WM, De Souza CR, Pek PP, Shahidah N, Ng YY, Arulanandam S, et al. myResponder smartphone application to crowdsource basic life support for out-of-hospital cardiac arrest: the Singapore experience. Prehosp Emerg Care. 2020;25(3):1–9; 10.1080/10903127.2020.1777233. 10.1080/10903127.2020.1777233 32497484

[39] Singapore Attorney-General’s Chambers. Human Organ Transplant Act (Chapter 131A). 2012. https://sso.agc.gov.sg/Act/HOTA1987. Accessed 23 Aug 2020.

[40] Ministry of Health. Understanding Human Organ Transplant Act (HOTA). 2013. https://www.liveon.gov.sg/docs/info_booklets/SO20870_Hota_english2013.pdf. Accessed 29 Aug 2020.

[41] Matesanz R, Domínguez-Gil B, Coll E, Mahíllo B, Marazuela R (2017). How Spain reached 40 deceased organ donors per million population. Am J Transpl..

[42] Today Singapore. Singapore needs more organ donors. 2016. https://www.todayonline.com/commentary/singapore-needs-more-organ-donors.

[43] The Straits Times. Organ donations remain low despite changes to law. 2016. https://www.straitstimes.com/singapore/organ-donations-remain-low-despite-changes-to-law. Accessed 19 Aug 2020.

[44] Oh YZ, Lee CT, Lim AT, Tong KL. Sports-related sudden cardiac deaths in Singapore – an eleven-year review. Ann Acad Med Singap 2019;48(5):156–160. 31210253

[45] Miñambres E, Rubio JJ, Coll E, Domínguez-Gil B (2018). Donation after circulatory death and its expansion in Spain. Curr Opin Organ Transplant.

[46] Ortega-Deballon I, Rodríguez-Arias D (2018). Uncontrolled DCD: when should we stop trying to save the patient and focus on saving the organs?. Hast Cent Rep.

